# Pan-Genome Analysis of Brazilian Lineage A Amoebal Mimiviruses

**DOI:** 10.3390/v7072782

**Published:** 2015-06-26

**Authors:** Felipe L. Assis, Leena Bajrai, Jonatas S. Abrahao, Erna G. Kroon, Fabio P. Dornas, Kétyllen R. Andrade, Paulo V. M. Boratto, Mariana R. Pilotto, Catherine Robert, Samia Benamar, Bernard La Scola, Philippe Colson

**Affiliations:** 1 Instituto de Ciências Biológicas, Departamento de Microbiologia, Laboratório de Vírus, Universidade Federal de Minas Gerais, Belo Horizonte, 31270-901 Minas Gerais, Brazil; E-Mails: felipelopesassis@gmail.com (F.L.A.); jonatas.abrahao@gmail.com (J.S.A.); ernagkroon@gmail.com (E.G.K.); fabiopiod154@gmail.com (F.P.D.); ketyllen@yahoo.com.br (K.R.A.); pvboratto@gmail.com (P.V.M.B.); 2 Unité de Recherche sur les Maladies Infectieuses et Tropicales Emergentes (URMITE) UM63 CNRS 7278 IRD 198 INSERM U1095, Aix-Marseille Univ., 13385 Marseille, France; E-Mails: leenaasd@gmail.com (L.B.); catherine.robert@univ-amu.fr (C.R.); benamarsamia@yahoo.fr (S.B.); bernard.la-scola@univ-amu.fr (B.L.S.); 3 Department of Biochemistry, Faculty of Science, King Abdulaziz University, 21589 Jeddah, Saudi Arabia; 4 Centro de Ciências Biológicas, Departamento de Microbiologia e Parasitologia, Laboratório de Virologia Aplicada, Universidade Federal de Santa Catarina, Florianópolis, 88040-900 Santa Catarina, Brazil; E-Mail: maryrp@gmail.com; 5 Institut Hospitalo-Universitaire (IHU) Méditerranée Infection, Assistance Publique-Hôpitaux de Marseille, Centre Hospitalo-Universitaire Timone, Pôle des Maladies Infectieuses et Tropicales Clinique et Biologique, Fédération de Bactériologie-Hygiène-Virologie, 13385 Marseille, France

**Keywords:** *Mimiviridae*, Samba virus, pan-genome, mimivirus, *Megavirales*, Amazonia virus, oyster virus, Kroon virus, genomics

## Abstract

Since the recent discovery of Samba virus, the first representative of the family *Mimiviridae* from Brazil, prospecting for mimiviruses has been conducted in different environmental conditions in Brazil. Recently, we isolated using *Acanthamoeba* sp. three new mimiviruses, all of lineage A of amoebal mimiviruses: Kroon virus from urban lake water; Amazonia virus from the Brazilian Amazon river; and Oyster virus from farmed oysters. The aims of this work were to sequence and analyze the genome of these new Brazilian mimiviruses (mimi-BR) and update the analysis of the Samba virus genome. The genomes of Samba virus, Amazonia virus and Oyster virus were 97%–99% similar, whereas Kroon virus had a low similarity (90%–91%) with other mimi-BR. A total of 3877 proteins encoded by mimi-BR were grouped into 974 orthologous clusters. In addition, we identified three new ORFans in the Kroon virus genome. Additional work is needed to expand our knowledge of the diversity of mimiviruses from Brazil, including if and why among amoebal mimiviruses those of lineage A predominate in the Brazilian environment.

## 1. Introduction

*Acanthamoeba polyphaga mimivirus* (APMV) was isolated in 1992. In 2003, it was identified as a nucleocytoplasmic large DNA virus (NCLDV) and the first member of the family *Mimiviridae* [1]. Before that identification, such morphological complexity, virion size (>400 nm), genome length (≈1.2 million base pairs (bp)) and extensive gene content (979 protein coding genes) had not been attributed to any virus. Moreover, functional studies revealed the presence of proteins unknown in other viral genomes, such as aminoacyl tRNA-synthetases and translation factors [2]. Since then, several studies have been conducted to determine how widespread and diverse this new viral family is. Currently, over 50 mimiviruses have been isolated from cooling tower water, freshwater and saltwater, soil, leech and clinical samples collected in England, France, USA, Chile, Brazil and Tunisia [3,4,5,6,7,8,9,10,11]. Additionally, DNA from these viruses was identified in the Sargasso Sea and other ocean samples during metagenomic studies [12,13,14].

The family *Mimiviridae* can be divided into a main group (Group 1), composed of mimiviruses infecting amoebal species, and a distantly related mimiviruses group (Group 2) comprised of *Cafeteria roenbergensis virus* (CroV; which infects a marine heterotrophic bi-flagellate) [15], organic lake phycodnaviruses and *Phaeocystis globosa* virus, as recently refined by phylogenomic studies [16]. Mimiviruses infecting amoebal hosts can be divided into three lineages (A [Mimivirus], B [Moumouvirus] and C [*Megavirus chiliensis*]) according to phylogenies based on conserved core genes including, for example, family B DNA polymerase and ribonucleotide reductase encoding genes [8,15,17,18]. Unexpectedly, the discovery of mimivirus allowed the detection of a new type of virus able to infect giant viruses. These new viral entities were named virophages, in analogy to bacteriophage behavior [4]. Of significant importance is the growing body of evidence for the presence of mimiviruses in humans and the recent isolation of two such giant viruses from pneumonia patients [11,19].

**Figure 1:**
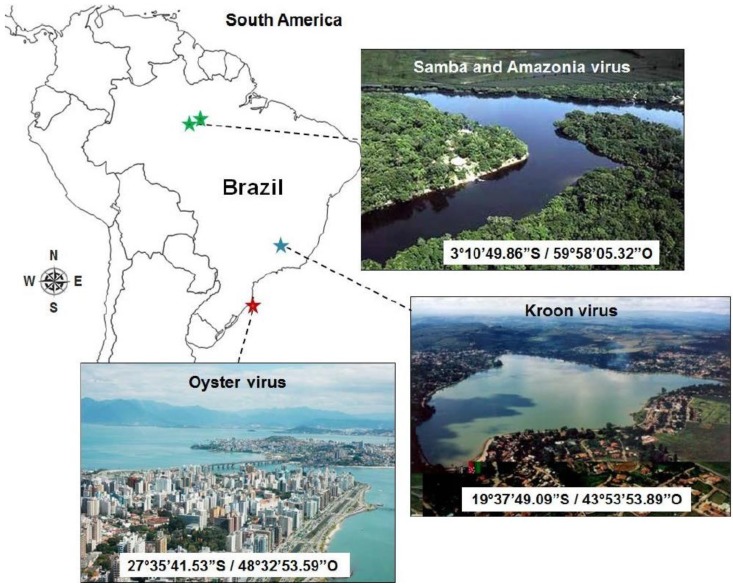
Geographical location, coordinates, and biome representation for each Brazilian mimivirus isolate.

Recently, a new member of lineage A of mimiviruses, named Samba virus (SMBV), was isolated in the Brazilian Amazon forest from a water sample collected in 2011 from the surface of Negro River (3°6′ S 60°1′ W) [8]. A new virophage, named Rio Negro virus (RNV), was also isolated in association with SMBV. SMBV was biologically and molecularly characterized [8]. Its genome was partially (~98%) sequenced, revealing a G+C content of 27.0%. A total of 938 ORFs, ranging in size from 150 to 8835 bp, with an average size of 1002 bp, were predicted. The RNV characterization is in progress (unpublished data), but previous data indicate that it is very similar to the Sputnik virophage. SMBV was the first mimivirus ever isolated in Brazil, which raised the question of the diversity, distribution and specific features of giant viruses from different Brazilian ecosystems. Since then, our Brazilian team has been engaged in prospective studies for giant viruses in Brazil. These studies led to the detection of mimivirus DNA and neutralizing antibodies in serum samples of domestic and wild mammals from the Amazon region [20]. In addition, we isolated three new Brazilian mimiviruses from different environmental sources in different Brazilian regions (Figure 1). The present work describes the complete genome sequencing of these three viruses and a pan-genome analysis of Brazilian mimiviruses. Moreover, we performed an updated analysis of the SMBV genome and the phylogenetic clustering of Brazilian mimiviruses among other mimiviruses into the NCLDV group, or proposed order *Megavirales* [21].

## 2. Materials and Methods

### 2.1. Mimivirus Origin and Isolation

The viruses described in this work were isolated from: (A) Amazonia virus (AMAV): water sample from the Negro River, Amazon forest, in 2011; (B) Kroon virus (KROV): water sample from an urban lake in Lagoa Santa city, Minas Gerais State, in 2012; and (C) Oyster virus (OYTV): oyster samples farmed on the Atlantic coast, Florianópolis, Santa Catarina state, in 2013 [22] (Figure 1). The samples were cultured in *A. castellanii* monolayers and the isolated viruses were grown and purified as described previously [8].

### 2.2. Genome Sequencing and Assembly

The genomes were sequenced using the Illumina MiSeq instrument (Illumina Inc., San Diego, CA, USA) with the paired end application. The sequence reads were assembled *de novo* using ABYSS software [23] and the resulting contigs were ordered by the python-based CONTIGuator.py software [24]. Concurrently, the CLC_Bio software [25] was used for mapping-based genome assembly using the APMV genome (NC_014649.1) as reference. Draft genomes obtained by both strategies were mapped back to check the reads assembly and to close gaps. The best assemblages for each genome were kept, and the few remaining small gaps were closed by Sanger sequencing.

### 2.3. Genome Annotation

The gene predictions were performed using RAST (Rapid Annotation using Subsystem Technology) [26] and GeneMarkS [27] tools. Transfer RNA (tRNA) sequences were identified using the tRNAscan-SE tool [28]. The functional annotations were inferred by BLAST searches against the GenBank NCBI non-redundant protein sequence database (nr) (*e*-value < 1 × 10^−3^), the set of clusters of orthologous groups of proteins (COGs) of the NCLDVs (named NCVOGs) [16] and by searching specialized databases through the Blast2GO platform [29]. Finally, the genome annotation was manually revised and curated. The predicted ORFs that were smaller than 100 amino acids and had no hit in any database were ruled out. The ORFs longer than 100 amino acids without hits in any database (ORFans) were kept.

ORFan transcripts were detected by Sanger sequencing after extraction from a culture supernatant with the RNeasy Mini kit (Qiagen, Hilden, Germany). The TURBO DNA-Free kit (Ambion, Austin, TX, USA) was used to remove contaminating DNA from RNA preparations as checked by performing PCR with the HotStar TaqDNA polymerase (Qiagen) in absence of a reverse transcription step. Then, RNeasy Mini Elute clean up kit (Qiagen) and SuperScript VILO cDNA synthesis kit (Invitrogen, Carlsbad, CA, USA) were used. Finally, this cDNA template was amplified by PCR with the Hot Star Taq DNA polymerase (Qiagen) then sequenced using the following primers: K22A-F: 5′-TTTCAAACAAATGCAACGTAAAGT; K22A-R: 5′-ACGCTTATTTGAAAACAAACCAAA; K22B-F: 5′-CAATTCTTTCAAACAAATGCAACG; K22B-R: 5′-ACGCTTATTTGAAAACAAACCAAAA; K61A-F: 5′-AGCGCCATGGTGTCCAATAA; K61A-R: 5′-CGAGTGTATGGACAACCGGTAA; K61B-F: 5′-ACCGGTTGTCCATACACTCG; K61B-R: 5′-ATTTCAACCGGATTATTCTTGGG; K933A-F: 5′-ACCATGATGGATATCCGGTGG; K933A-R: 5′-TCAGGATGGATATTTGCCGTGT; K933B-F: 5′-TGGATATCCGGTGGATGAAGA; K933B-R: 5′-ATCAGGATGGATATTTGCCGT. In addition, we evaluated the expression of viral tRNA molecules into cells infected with Brazilian mimiviruses. Total RNA was extracted using the RNeasy kit (Qiagen), and reverse transcription was performed by using the MMLV reverse transcriptase (Promega, Madison, WI, USA), following the manufacturers recommendations. The cDNA was used in qPCR reactions using specific primers (Leucyl-tRNA: forward 5′-GGGATTCGAACCCACGACAT, reverse 5′-ATAAGCAAAGGTGGCGGAGT; Histidyl-tRNA: forward 5′-TTAGTGGTAGAACTACTGTTTGTGG, reverse 5′-TTTTCAAAAATGACCCGTACAGGAA; Cysteinyl-tRNA: forward 5′-ACAGTCAACTGGATCGTTAGC, reverse 5′-AGGATCGTATCAGAATTGAACTGA; Tryptophanyl-tRNA: forward 5′-GTGCAACAATAGACCTGTTAGTTTA, reverse 5′-ACCGGAATCGAACCAGTATCA) with SYBR Green PCR Master Mix (Applied Biosystems, Foster City, CA, USA). Reactions were carried out on the StepOne instrument (Applied Biosystem) and optimized. Relative gene expression was evaluated by using the 2-Delta-Delta *C*t method [30] and normalized to 18S ribosomal RNA and viral RNA helicase mRNA.

### 2.4. Comparative Genomic Analysis

The co-linearity between Brazilian mimiviruses was checked using MUMmer.3.23 [31] and MAUVE programs [32]. The Proteinortho tool [33] was used to define the *bona fide* orthologous genes shared among Brazilian mimiviruses and representatives of amoebal mimiviruses of lineages A–C, using the reciprocal best hits strategy with 1 × 10^−3^, 30% and 70% as thresholds for the BLASTp *e*-value, and identity and coverage of amino acid sequences, respectively. The OrthoMCL tool [34] was used to identify the paralog families among Brazilian mimivirus proteins.

### 2.5. Analysis of the Pan-Genome and Core Genome of Brazilian Mimiviruses

To estimate the size of the pan-genome of Brazilian mimiviruses, their predicted proteins were clustered by the BLASTclust program using an amino acid sequence identity of 30% and sequence coverage of 70% as the parameters. We also described the pan-genome size evolution by stepwise inclusion of each new virus annotation in the pairwise comparisons of the gene contents. The orthologous protein clusters encompassing at least one protein from each virus, obtained by the BLASTclust and Proteinortho5.pl programs, were taken into account to determine the core genome and strict core genome, respectively, of the mimivirus lineage A group.

### 2.6. Phylogeny

Protein sequences of Brazilian mimiviruses were aligned with those from the other giant viruses previously described in GenBank using the ClustalW program [35]. After visual analyses and manual curation of protein sequence alignments, the phylogeny reconstruction was performed using the maximum likelihood method implemented by MEGA5 software [36].

## 3. Results

### 3.1. Genome Annotation of Brazilian Mimiviruses

The genomes of AMAV (GenBank Accession no. KM982403), KROV (KM982402) and OYTV (KM982401) are double-stranded DNA molecules of 1,179,579, 1,221,932 and 1,200,220 bp, respectively. KROV presents the largest DNA genome among lineage A amoebal mimiviruses. Its genome is ~21, 42 and 40 kb larger than those from OYTV, AMAV and SMBV, respectively, and ~30 kb and 40 kb larger than those from Mamavirus and APMV, respectively. These genomes have a mean G+C content of 27%, similar to other mimiviruses. The genomes of AMAV and OYTV contain six regions encoding the tRNA molecules for leucine (3 sequences), histidine, cysteine and tryptophan amino acids, whereas the genome of KROV lacks the sequence encoding the tRNA for tryptophan. The assessment of tRNA gene expression was congruent with the genomic analyses, by detecting six tRNA molecules in SMBV and OYTV, whereas no tryptophanyl-tRNA expression was detected in KROV.

We predicted 979 ORFs for AMAV, 944 for KROV and 948 for OYTV, with ORF sizes ranging from 113 bp to 8835 bp (mean, 1046 bp). For all genomes, the predicted protein-encoding genes were evenly distributed on both positive and negative DNA strands (in ~51% of cases on the negative strand). A total of 35 clusters consisting of 125 paralogous proteins were identified in the KROV genome, 54 clusters consisting of 174 paralogous proteins were identified in the OYTV genome and 55 clusters consisting of 176 paralogous proteins were identified in the AMAV genome (Table 1). Although the KROV genome is the largest among the lineage A amoebal mimiviruses, it was predicted to encode fewer ORFs, including fewer paralogous proteins. Thus, approximately 13% of the KROV ORFs were comprised of paralogs, whereas this proportion was 18% for ORFs encoded by the OYTV, AMAV and SMBV genomes.

**Table 1:** Summary of results for genome annotation from Brazilian mimiviruses.

Brazilian Mimivirus	Viral Source/Year of Isolation	Genome Size (bp)	G+C %	Number of ORFs	Number of ORFans	Number of APMV Orthologs	Number of Paralogous Proteins/Clusters	tRNAs
Samba virus	Negro River water, Amazon forest/2011	1,181,380	28.0	971	0	916	178/58	6
Amazonia virus	Negro River water, Amazon forest/2011	1,179,579	27.9	979	1 *	905	176/55	6
Oyster virus	Oyster farmed in Atlantic cost, Florianopolis, SC/2013	1,200,220	27.9	948	1 *	864	174/54	6
Kroon virus	Urban lake water, Minas Gerais state/2012	1,221,932	27.5	944	3	769	125/35	5

APMV, *Acanthamoeba polyphaga* mimivirus; bp, base pair; ORF, open reading frame; SC: Santa Catarina state. * ORFans with no hit with the following criteria: *e*-value: <1 × 10^−3^; similarity: >30%; coverage: >50%.

**Figure 2:**
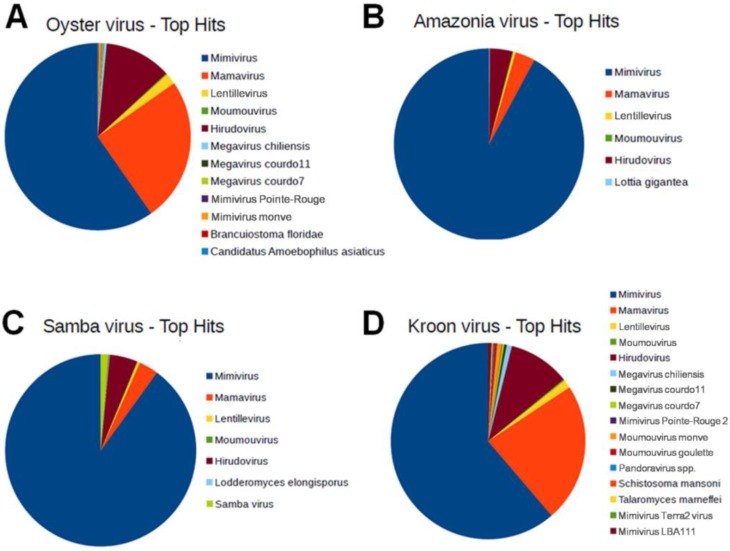
Graphical distribution of the best BLAST hits for Brazilian mimivirus gene contents. The analysis was performed using BLASTp algorithm searching with the predicted ORFs from Brazilian mimiviruses against the NCBI GenBank non-redundant (nr) protein sequence database using the java-based free software Blast2GO [29]. (**A**) Oyster virus; (**B**) Amazonia virus; (**C**) Samba virus; and (**D**) Kroon virus. The *Acanthamoeba polyphaga* mimivirus was the predominant target for hits from all Brazilian mimiviruses (896, 588 and 577 best hits to AMAV, OYTV and KROV, respectively), with an average of 76% of the hits.

The functional annotation revealed that ~50% of the ORFs in Brazilian mimivirus genomes were hypothetical proteins, *i.e.*, without a defined function. For all the Brazilian mimivirus genomes, the best hits were most frequently proteins from APMV, followed by those from Mamavirus (Figure 2). These viruses belong to lineage A of the amoebal mimiviruses. One ORF in the AMAV genome, three ORFs in the KROV genome and three ORFs in the OYTV genome had no hit against the NCBI nr protein sequence database or the Blast2GO platform. To confirm whether these genes were *bona fide* ORFans, their nucleotide sequences were submitted to a BLASTn search against the GenBank nucleotide sequence database (nt) of the NCBI. For AMAV, the putative ORFan R931 had hits with other lineage A mimiviruses (coverage of 49%; similarity of 100%; *e*-value of 1 × 10^−20^ or less). In contrast, the three putative KROV ORFans (L22, L61 and R933 genes) had no significant hits. Transcripts for these three ORFans were detected by Sanger sequencing. For OYTV’s putative ORFans, significant hits against lineage A mimiviruses were only obtained for R123 and L733 genes. For ORFan L25, a hit with an *e*-value of 0.63, coverage of 83% and identity of 26% was observed against Lausannevirus, a giant amoebal virus from the family *Marseilleviridae*. Whether these putative ORFans were shared by Brazilian mimiviruses was determined by BLASTn analyses. The putative ORFan of AMAV was shared by the KROV and OYTV genomes, whereas ORFans from KROV were not found in any other Brazilian mimivirus genomes. The OYTV putative ORFans L733 and R123 were shared by KROV and AMAV, whereas ORFan L25 was exclusive to the OYTV genome.

### 3.2. Samba Virus Genome Update

For this new genome announcement, the SMBV genome was re-sequenced using Illumina Deep Sequencing Technology. The assembled genome obtained consisted of a double-stranded DNA molecule of 1,181,380 bp with a G+C content of 28%, similar to that of other mimivirus isolates. Although shorter (30,300 bp less) than in the first report, the SMBV genome was predicted to encode 971 ORFs (33 more ORFs than in the first report), ranging in size from 113 to 8834 bp, with a mean size of 1068 bp. The ORF density was 0.82 genes/kb, with a coding density of 87.9%. The genome of SMBV was predicted to encode 142 cellular metabolism-associated proteins and up to 180 proteins involved in general metabolic processes, in addition to some regulatory proteins, suggesting a certain level of autonomy for the virus, even as an obligate intracellular parasite. Among the predicted ORFs, 45% were hypothetical proteins, and 85% showed a best hit against APMV. We identified four sequences encoding aminoacyl-tRNA synthetase, a landmark of mimiviruses because no other virus encodes such proteins. Moreover, we were able to identify six tRNA sequences: three cognates to leucine, and one to histidine, cysteine or tryptophan. The G+C content of these tRNA sequences was 48.6%, far greater than that for the entire genome.

All the predicted ORFs matched orthologs in the NVCOG database with an average similarity of 93.7%; thus, new ORFans were not predicted. A total of 58 clusters consisting of 179 paralogous proteins were identified in the SMBV genome, similar to that detected in the APMV and Hirudovirus genomes. The reciprocal best hit analysis identified 917 *bona fide* orthologous proteins between SMBV and APMV. The same analysis identified 339 *bona fide* orthologous proteins shared with Moumouvirus (lineage B of amoebal mimiviruses) and 485 shared with *Megavirus chilensis* (lineage C).

### 3.3. Comparative Genomics of Lineage A Mimiviruses

The genome sequence of SMBV was most closely related to APMV, with a similarity level of 99% (and a coverage of 100%), followed by AMAV (similarity: 99%, coverage: 99%), OYTV (similarity: 99%, coverage: 97%) and KROV (similarity: 94%, coverage: 90%). The same tendency was observed among Brazilian mimiviruses and other lineage A mimiviruses, such as Mamavirus (JF801956.1), Terra2 virus (NC_023639.1) and Hirudovirus (KF493731.1) (data not shown). Among the mimi-BR, SMBV, AMAV and OYTV genomes had similarity values ranging from 97% to 99%, whereas KROV had similarity values between 90%–91% with the other mimi-BR.

**Figure 3:**
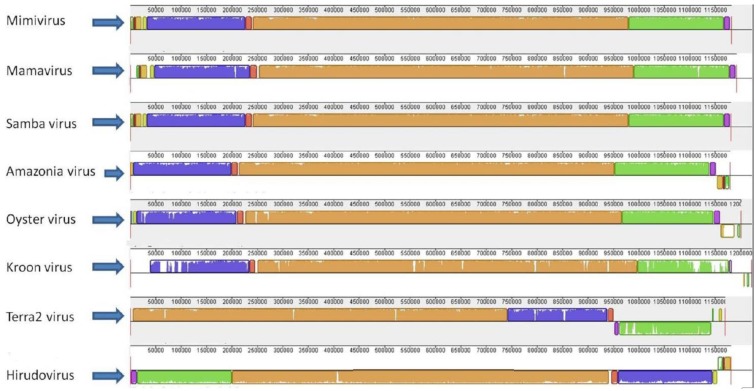
Genome alignment of lineage A amoebal mimiviruses showing their genome architecture and synteny. The genome alignment and schematic were obtained using the Mauve software package [32].

The gene synteny analysis showed that mimi-BR genomes share a similar architecture with those from the other lineage A mimiviruses, with some reassortments at their extremities, primarily compared to the Terra2 virus and Hirudovirus (Figure 3). While analyzing the mimi-BR alignment, we identified short fragments at the 5′ extremity of the SMBV genome that were related to fragments at the 3′ extremities of the OYTV and AMAV genomes but inverted in orientation. Furthermore, we observed the presence of a large region (~30,000 bp) at the 5′ extremity of the KROV genome that had no similarity with any region in the SMBV, OYTV and AMAV genomes. This singular region in the 5′ extremity of the KROV genome was predicted to encode 25 ORFs, of which 16 (66%) encode ankyrin-like proteins (Figure 3). These ORFs seem to be comprised of fragmented and/or intron-containing genes, besides some duplicated genes, resembling what was described previously at the 5′ extremity of the Mamavirus genome [37]. KROV also presents differences in some gene structure, including the major capsid protein-encoding gene (APMV L425), which might be involved in a particular splicing process.

For all mimi-BR genomes, the paralogous genes were predominantly distributed towards both extremities of each genome (59% on average), with few occurrences in the central regions (average of 7%) where nevertheless, as for the case of the KROV genome, we observed genes flanked by paralogs. Although the majority of these paralogs were co-localized, in some cases, genes from paralogous gene pairs were found at different genome extremities and in inverted orientations, as previously described for Mimivirus [38].

**Figure 4:**
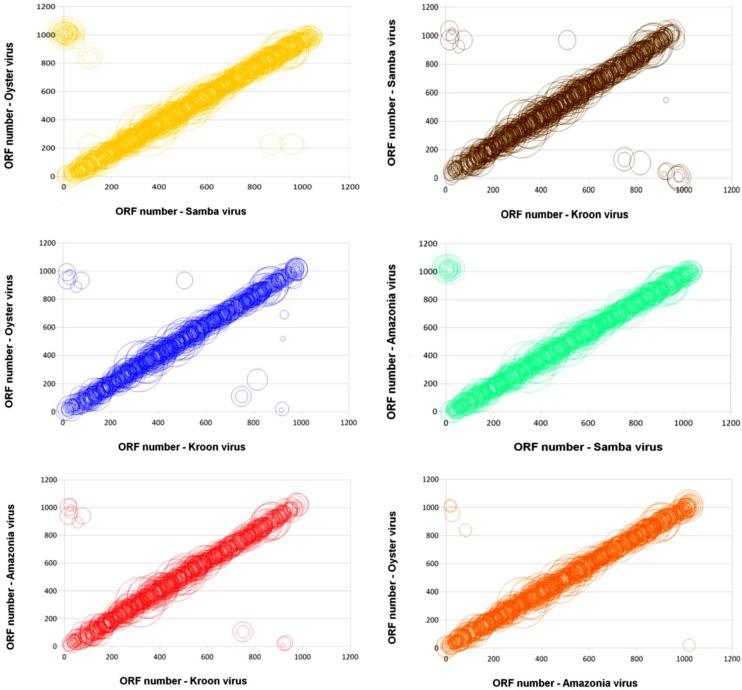
Genomic dot-plots based on a BLASTp analysis for pairs of Brazilian mimiviruses. Each circle shows a pair of orthologous proteins found in each pair of Brazilian mimiviruses. The diameters of the bubbles are proportional to the BLASTp similarity scores, and their positions are relative to the position of each pair in the genome.

The reciprocal best hits (RBH) analyses of protein contents corroborated with genome synteny analyses. The SMBV shared 950, 892 and 781 RBHs with AMAV, OYTV and KROV, respectively. AMAV and OYTV shared 886 RBHs, whereas KROV shared 784 and 785 RBHs with AMAV and OYTV, respectively. Moreover, these orthologous genes shared a high level of colinearity when compared with their counterparts from other mimi-BR genomes (Figure 4).

### 3.4. Pan-Genome and Core Genome Analysis

**Figure 5:**
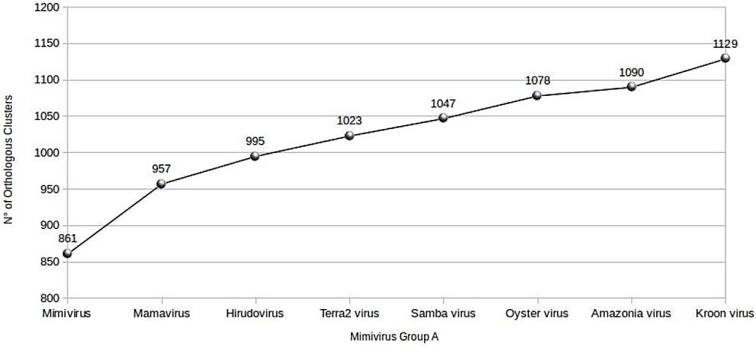
Evolution of the pan-genome size of amoebal mimiviruses of lineage A.

Using the BLASTclust program, an increasing pan-genome size was noted to each genome annotation of the new mimiviruses, to finally obtain a total of 1129 clusters, which we identified as the pan-genome of the lineage A mimiviruses infecting amoeba (Figure 5). Taking into account only the pan-genome of the Brazilian mimiviruses, a total of 3877 proteins were grouped into 974 clusters of orthologous proteins. The size of the core genome of mimiviruses of lineage A, including Brazilian mimiviruses, was calculated using all clusters of orthologous proteins and *bona fide* orthologous proteins created by BLASTclust and Proteinortho5.pl programs, respectively. These two approaches delineated a core genome comprised of 597–644 genes and similar curves for the size of the core genome of lineage A amoebal mimiviruses, the number of orthologs shared by all of these viruses decreasing with each new genome annotation.

### 3.5. Phylogeny

**Figure 6:**
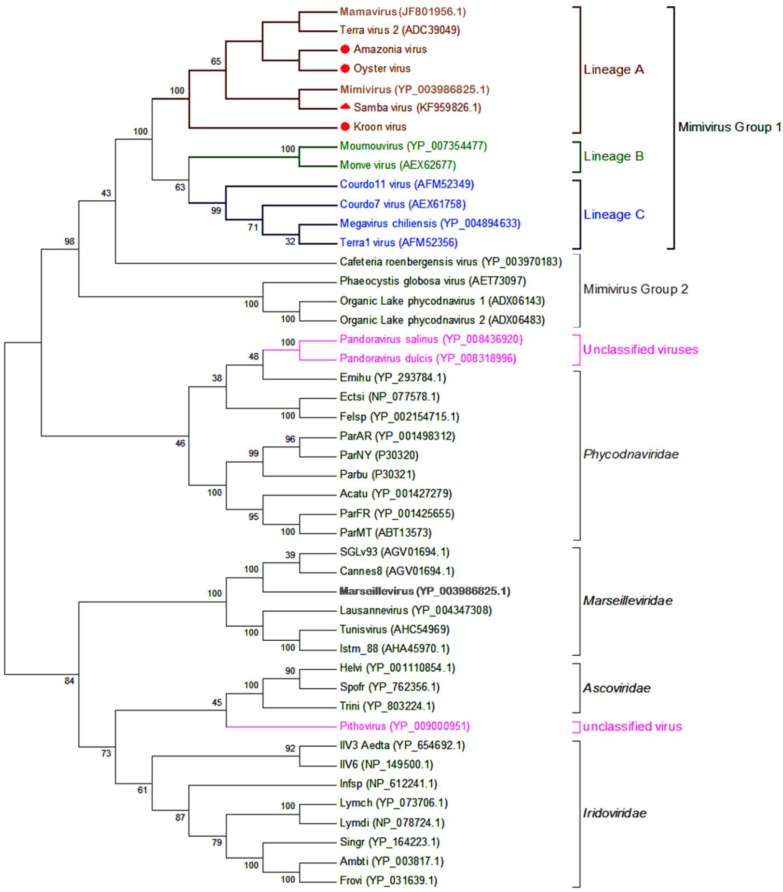
Phylogenetic reconstruction of Brazilian mimiviruses and other megaviruses based on family B DNA polymerase. A phylogenetic tree was generated using MEGA5 software with the Maximum likelihood method. The percentage of trees using 1000 bootstrap replicates in which the associated taxa clustered together is shown next to the branches. Brazilian mimiviruses, highlighted by red markers, are clustered with members of lineage A amoebal mimiviruses. The circles indicate new viruses; the triangle indicates the previously reported Brazilian mimivirus. For each sequence, the GenBank gene identification numbers are indicated. The branches were identified by brackets and family names. Branches corresponding to lineages of amoebal mimiviruses are differentiated by colors. Currently unclassified viruses are highlighted in pink.

The phylogenetic tree based on family B DNA polymerase showed that all Brazilian mimiviruses were clustered with other lineage A mimiviruses in the major group of the family *Mimiviridae* (Figure 6). It is worth mentioning that OYTV and AMAV were more closely related to each other than to other lineage A members, as were SMBV and APMV. Congruent with results from comparative genomics, KROV was more divergent from the other Brazilian mimiviruses, although robustly clustered with other lineage A mimiviruses.

## 4. Discussion

Since the description of the original strain of Mimivirus in 2003, eight mimiviruses infecting *Acanthamoeba* spp. have been isolated and biologically and molecularly characterized. Here, we have described the genomic characterization of three new amoebal mimiviruses isolated from different environments in Brazil and the re-analysis of the genome of SMBV, the first *Mimiviridae* isolate from this country. In particular, we studied the gene arsenal and genome architecture of these viruses and their relationship with other members of this viral family. As for other mimivirus genomes, the majority of the ORFs in Brazilian mimivirus genomes were ORFans or putative genes encoding hypothetical proteins, and their annotation was based on similarity with previously described mimiviral genomes. Thus, most of these ORFans are family ORFans, which means that they have no known ortholog in public sequence databases apart from other mimiviral genomes [39]. Hence, the function, significance and evolutionary relationship remain to be deciphered for a significant proportion of putative genes in these mimiviral genomes.

As previously described [8], the SMBV genome is closely related to APMV and has high similarity with other Brazilian mimiviruses such as OYTV and AMAV. The analysis of the clusters of orthologous proteins among these three viruses particularly highlights their proximity. However, although more closely related to Brazilian mimiviruses than to other lineage A mimiviruses, the KROV isolate showed singular features compared to SMBV, OYTV and AMAV. For example, this virus has the largest genome among amoebal mimiviruses of lineage A. Nonetheless, the KROV genome encodes fewer ORFs, which comprise fewer paralogs than other lineage A amoebal mimiviruses, but increases the pan-genome size for these mimiviruses. In addition, the KROV genome is lacking the cognate tRNA to tryptophan and is predicted to encode three new genuine ORFans, unknown in any other organism.

It has been suggested that mimiviruses from different geographical areas may be closely related [6]. Examples include *Megavirus chilensis*, isolated from coastal seawater in Chile, and Courdo11 virus, isolated from freshwater samples in Southeastern France [5,40]. Brazilian ecosystems have a high level of complexity, and the majority of these ecosystems remain to be investigated. Previous results indicated that mimiviruses are common in aquatic environments in Brazil, and the isolation and analysis of new mimiviruses from this country contribute to our knowledge of the diversity and evolution of this viral family. The Brazilian mimiviruses studied here were isolated from different environments, including fresh- and saltwater, but were all clustered in the same lineage of amoebal mimiviruses that includes Mimivirus, Mamavirus and Terra2 viruses isolated in England and France from freshwater or soil [2,4,41]. The presence of mimiviruses in animals and humans is also of interest. Recently, our Brazilian team detected mimiviruses in serum samples from wild and domestic animals [20], while other studies have suggested a role for mimiviruses in pneumonia, which was strengthened by the recent isolation of two mimiviruses in patients with atypical unexplained pneumonia [11,19]. Overall, the exploration of the diversity of mimiviruses in different environments may help to solve many questions about the mimivirus life cycle and their clinical importance.
